# Supplementary material for: A Universal Bleeding Risk Score in Native and Allograft Kidney Biopsies: A French Nationwide Cohort Study
Source: J Clin Med. 2023 May 17;12(10):3527. doi: 10.3390/jcm12103527 (PMC10219527; doi:10.3390/jcm12103527)

Supplemental Material

Figure S1: Rate of bleeding using the previous score (validated for native kidneys)

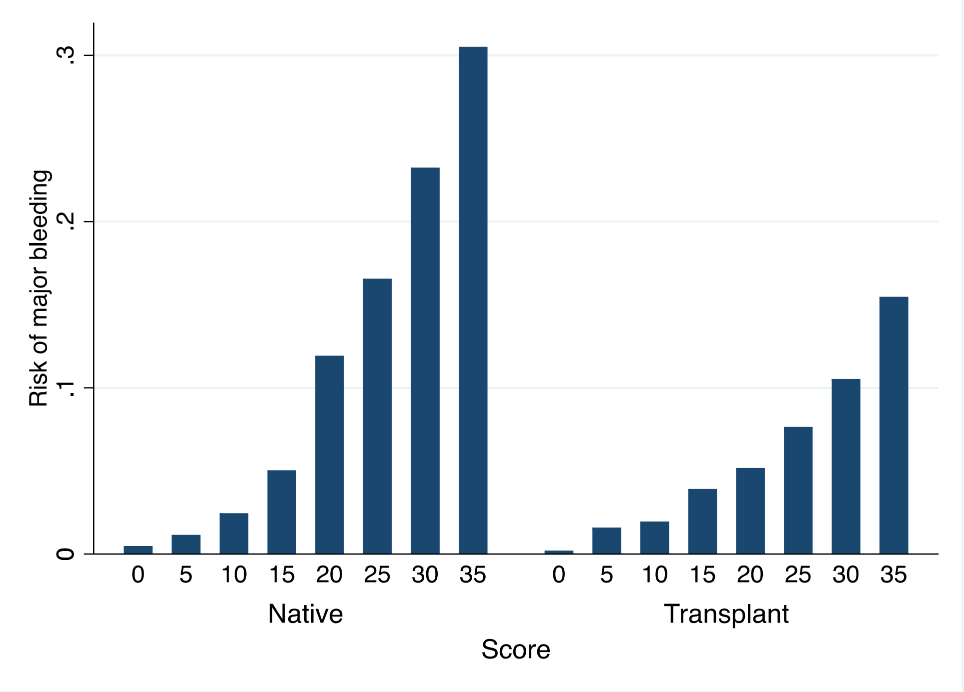

Figure S2: AUC ROC curve of the previous score (24) (initially validated in patients with percutaneous kidney biopsies) applied to kidney transplant recipients

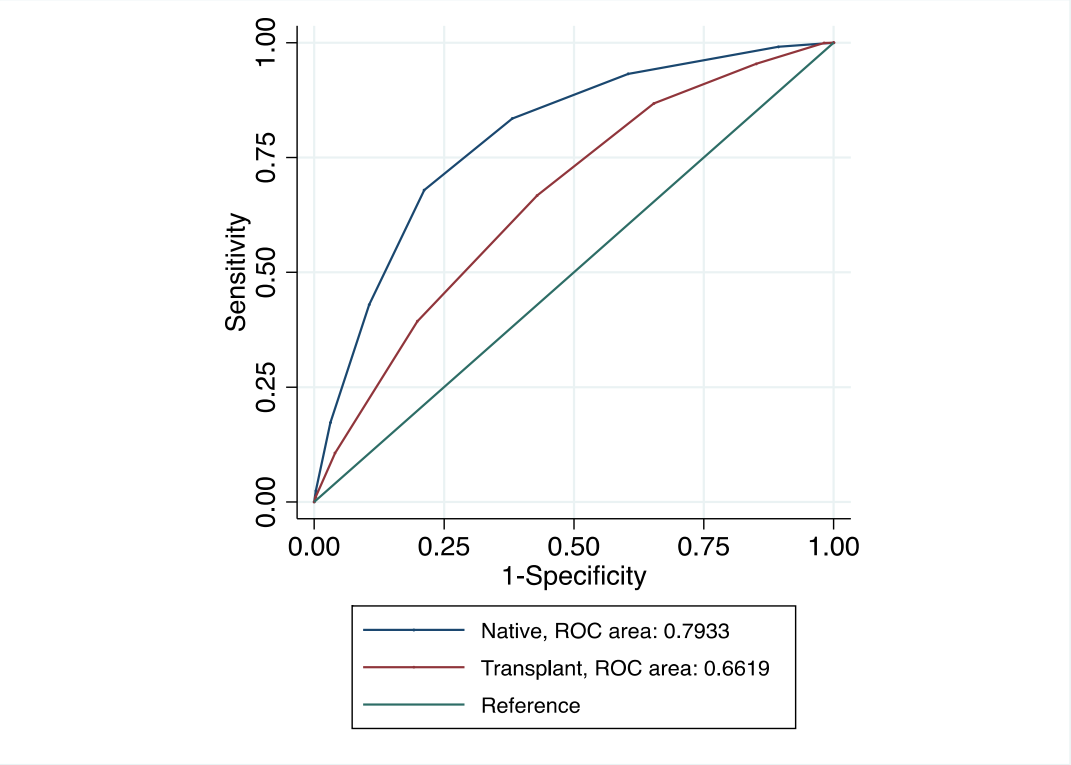

Supplement: Supplementary file 1 [file jcm-12-03527-s001.zip › jcm-2333931-supplementary.pdf]
